# Audio-visual interactions uniquely contribute to resolution of visual conflict in people possessing absolute pitch

**DOI:** 10.1371/journal.pone.0175103

**Published:** 2017-04-05

**Authors:** Sujin Kim, Randolph Blake, Minyoung Lee, Chai-Youn Kim

**Affiliations:** 1 Department of Psychology, Korea University, Seoul, Korea; 2 Department of Psychological Sciences, Vanderbilt Vision Research Center, Vanderbilt University, Nashville, Tennessee, United States of America; 3 Department of Brain and Cognitive Sciences, Seoul National University, Seoul, Korea; Goldsmiths University of London, UNITED KINGDOM

## Abstract

Individuals possessing absolute pitch (AP) are able to identify a given musical tone or to reproduce it without reference to another tone. The present study sought to learn whether this exceptional auditory ability impacts visual perception under stimulus conditions that provoke visual competition in the form of binocular rivalry. Nineteen adult participants with 3–19 years of musical training were divided into two groups according to their performance on a task involving identification of the specific note associated with hearing a given musical pitch. During test trials lasting just over half a minute, participants dichoptically viewed a scrolling musical score presented to one eye and a drifting sinusoidal grating presented to the other eye; throughout the trial they pressed buttons to track the alternations in visual awareness produced by these dissimilar monocular stimuli. On “pitch-congruent” trials, participants heard an auditory melody that was congruent in pitch with the visual score, on “pitch-incongruent” trials they heard a transposed auditory melody that was congruent with the score in melody but not in pitch, and on “melody-incongruent” trials they heard an auditory melody completely different from the visual score. For both groups, the visual musical scores predominated over the gratings when the auditory melody was congruent compared to when it was incongruent. Moreover, the AP participants experienced greater predominance of the visual score when it was accompanied by the pitch-congruent melody compared to the same melody transposed in pitch; for non-AP musicians, pitch-congruent and pitch-incongruent trials yielded equivalent predominance. Analysis of individual durations of dominance revealed differential effects on dominance and suppression durations for AP and non-AP participants. These results reveal that AP is accompanied by a robust form of bisensory interaction between tonal frequencies and musical notation that boosts the salience of a visual score.

## Introduction

Perception–the sensory based experience of objects and events in our environment—seems to transpire automatically and effortlessly, but that impression is illusory [1]. In fact, perception is the culmination of multiple neural operations carried out within a network of specialized cortical and subcortical areas distributed throughout the brain. Moreover, the successful performance of those distributed neural operations would be stifled by the inherent ambiguity in the sensory information that launches the whole chain of events if it were not for the intervention of prior knowledge of likely possibilities and for the coalescence of information from the different sensory modalities [2]. It is this latter contribution to perception–multisensory interaction–that is the focus of the work described in this paper.

For a number of years, investigators have been capitalizing on perceptual ambiguity to study how the different senses combine information to resolve confusion and conflict. Among the phenomena deployed in those studies, binocular rivalry has figured importantly [3]. Rivalry occurs where two dissimilar monocular images are presented to corresponding retinal locations of the two eyes: rather than blending into a stable binocular impression, the two images compete for visual dominance, meaning that people experience spontaneous, unpredictable alternations in perceptual awareness of the two dissimilar images [4–6]. This competition between two conflicting perceptual outcomes is influenced by visual factors such as contrast [7, 8], motion [9], and complexity [10], but non-visual factors too are very important determinants of the dynamics of rivalry [11]. Among those factors are sensory signals supplied by other modalities including olfaction [12], touch [13, 14] and hearing [15, 16]. Our laboratories have recently explored auditory influences on visual rivalry using music as the common thread tying together the two [17]. Specifically, we found that a visual musical score accompanied by an auditory melody predominated over a drifting grating during binocular rivalry, but only for individuals who can read music. That finding was not so surprising, but this aspect of the results was: melodic scores predominated more for trained musicians when the accompanying auditory melody was congruent with the visual score compared to when it was incongruent. That robust audio-visual interaction revealed that the resolution of visual competition can be influenced by high-level, symbolic representations, namely melodic structure and musical notation.

Inspired by that finding, the current study sought to examine bisensory congruence based on the *pitch* relation between the notes heard and the notes visualized: for “pitch-congruent” audio-visual pairings, the actual pitches comprising the auditory melody exactly matched the notes comprising the visual score, and for “pitch-incongruent” pairings the auditory melody was transposed by one semitone upward or downward relative to the notes comprising the visual score. We want to stress that for the pitch-incongruent condition, the melody itself *was* congruent. The key to our study was the individual difference among participants in terms of their ability to perceive pitch. Specifically, some of our participants possessed absolute pitch (AP), i.e., the immediate and effortless realization of the actual musical notes being played [18]. This rare condition indicates an ability to identify or produce the pitch of any tones without referring to another tone [19]. Others among our participants, while experienced musicians, did not have AP. Because we used subtle auditory/visual pitch differences in the pitch-incongruent condition, we expected that only participants with AP–and not those without AP–would sense the difference between those two conditions. Would that difference experienced by people forming those two groups differentially impact the visual selection of the score, even though the auditory stimuli had nothing to do with performance of the visual task? In this paper we report results providing an affirmative answer to that question. To set the stage for our study, the following paragraphs provide a précis of AP and why it affords a unique opportunity to examine the subtlety of bisensory interactions in binocular rivalry.

AP does not necessarily entail exceptional pitch acuity per se but rather a remarkable ability for categorizing and identifying tone pitch [19]. Moreover, AP is accompanied by unusually robust neural registration of low-level acoustic features. Marked neural activation [20] and increased connectivity [21–23] have been documented in the early auditory regions of the brains of people with AP, including the left superior and middle temporal regions. At the same time, people with AP apparently do not rely on auditory tonal working memory during a task requiring comparison of an individual tone with other tones in succession, as indicated by their ability to identify each tone based solely on its acoustic content and by the absence or reduction of a strong P300 event-related potential (ERP) in AP people when they hear an oddball tone among repeating tones [24–26]. It should be noted however, that attenuated P300 amplitude has not been found in other studies that have measured auditory pitch discrimination [27–30]. In addition, the right inferior frontal lobe, ordinarily involved in memory for pitch, is not involved when people with AP engage in a task requiring them to judge whether a pair of successive tones produced a major interval or a minor interval. Such a task would require pitch memory if the AP participants were, in fact, utilizing relative pitch (RP) to perform the task, but apparently they were not [31].

It is natural to surmise that possession of AP would be beneficial to a musician, and indeed a number of renowned composers including Bach, Beethoven, Mozart, and Toscanini possessed AP [32]. Performing musicians, too, who have AP find this ability useful when it comes to detecting pitch errors, including such errors in atonal music where there is no tonic pitch to serve as a reference [33]. AP is also beneficial in sight-reading musical scores because it helps to reconstruct musical passages from the visual musical notations. Keyboard players with AP, for example, may not need to look down frequently at their hands when sight-reading an unfamiliar score, since they can readily hear when they play incorrect notes.

At the same time, however, AP can be bothersome and, even, disruptive. Musicians with AP experience difficulty with transposed melodies in which pitch relations are preserved but absolute pitches are shifted to a different key relative to the compositionally defined original key: a transposed melody is discernibly different from its original counterpart [34]. Thus when listening to a transposed melody, they may “feel extremely uncomfortable” and may “complain that it is in the wrong keys or sounds out of tune” [35]. Consequently, musicians with AP are impaired when asked to judge whether or not an auditory melody and a transposed version are identical, a judgment easily made by musically trained people without AP [35, 36]. Musicians with AP also have trouble producing transpositions in pitch from the actual score when singing or playing an instrument [37, 38]. They can also experience agitation when listening to a melody transposed from the actual score, which is played in the “wrong” key to their ear [19, 35, 39]. When listening to transposed melodies, musicians with AP find it difficult to judge whether a visually presented melody and an auditorily presented transposed melody retain the same melodic line [35, 36]. It has been noted that these kinds of interference effects resemble the Stroop effect [33, 40], where incongruent pairing of a color word and the ink color in which that word was printed (e.g., “RED” printed in blue) slows down reporting the ink color (blue) due to the interference of the meaning of the word (red).

Intrigued by these mixed blessings associated with AP, we sought to learn whether possessing AP would impact binocular rivalry dynamics, a putatively visual phenomenon having nothing explicitly to do with an auditory cue as subtle as musical pitch.

## Methods

### Participants

Nineteen undergraduate and graduate students including two of the authors (SK and ML) participated in the experiment. Most participants were recruited from Korea University and Seoul National University with only a few exceptions. All of them could read musical scores, and had normal or corrected-to-normal vision with good stereopsis. They gave written informed consent approved by Korea University’s Institutional Review Board (1040548-KU-IRB-13-149-A-2). Participants were divided into two groups, either AP (N = 9, 8 female, 21.11 ± 1.76 (SD) years in age) or non-AP (N = 10, 9 female, 22.5 ± 2.59 (SD) years in age), based on their performance on an AP test described below. It is worth noting that individuals with extensive musical training could be categorized as non-AP if they produced a low score on the test; indeed, many of our non-AP participants had years of musical training though not as extensive as that of AP participants (average training of 13 ± 4.87 (SD) years for AP; 9 ± 5.5 (SD) years for non-AP). We discuss later, however, that our results are unlikely to be attributable to years of musical training.

### Apparatus and stimuli

**Fig 1:**
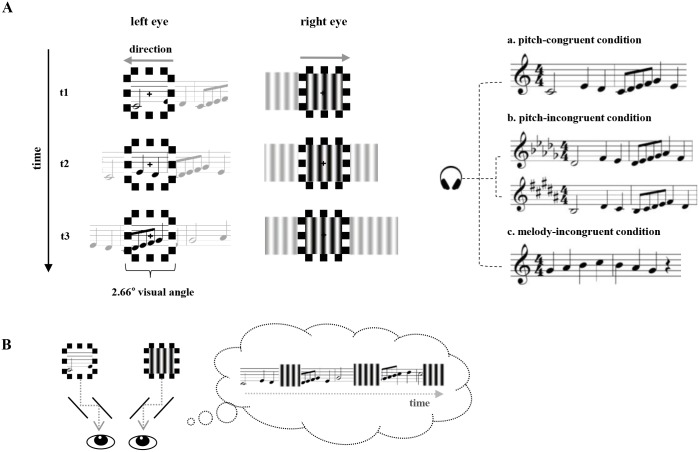
Stimulus presentation and experimental conditions. (A) Audio-visual stimuli and experimental conditions. (Left) Visual stimuli viewed through a mirror stereoscope by a participant. One eye viewed a vertical grating scrolling from the left to the right, and the other eye viewed one of six scores scrolling from right to left. Stimulus-eye pairings were counterbalanced across trials. The score here is sparsely sampled over time for illustration purpose. In the actual experiment, the score scrolled smoothly through the viewing window. Both visual stimuli were seen within a checkerboard-textured frame to facilitate stable binocular alignment. Participants were instructed to maintain fixation on the small spot at the center of the two dichoptic images. (Right) Given the visual stimuli exemplified on the left side, a melody matching the visual score (“pitch-congruent”), matching the melody of the score but transposed (“pitch-incongruent”), or not matching the score (“melody-incongruent”) was concurrently presented as an auditory stimulus. The auditory stimulus for a melody-incongruent condition shown on the right is one example among the possible five remaining scores not used as a visual stimulus. (B) Simulation of changes in perceptual dominance experienced during binocular rivalry; these changes would be characteristic of a short excerpt from a complete tracking trial, which would contain many more spontaneous alternations in rivalry state over the entire duration of a trial. In addition, there would also occur brief periods during which parts of both rival stimuli would be visible (mixture states are not illustrated here).

All stimuli were created and presented with MATLAB using Psychophysics Toolbox-3 [41, 42]. A vertical grating moving in one direction and one of six musical scores scrolling in the opposite direction were presented dichoptically through a mirror stereoscope (Fig 1A and 1B). Each of the two rival targets subtended 1.9° x 1.9° visual angle, and included a small fixation point subtending 0.39° x 0.39° visual angle located in the center of the target. To maintain stable binocular eye alignment, the dichoptically viewed score and the grating were surrounded by checkerboard-textured frames subtending 2.66° x 2.66° visual angle. The vertical, sinusoidal grating was 2.1 c/deg in spatial frequency and 60% Michaelson contrast; the grating drifted smoothly from left to right at 1.6°/s.

The visual scores and auditory files were created with the music notation software Sibelius (Avid Technology, Inc., Burlington, MA). Scores were the same ones used in our previous study [17], excerpted from vocal scores. Each score image comprised a series of dark gray notes appearing against white background. For each score, three auditory sequences were generated. For one of those sequences, the auditory melody matched the notation of the visual score in both pattern and pitch (i.e., pitch-congruent sequence). For a second sequence, the auditory and visual melodies were congruent but the auditory sequence was *transposed* from the original score one-semitone higher or one-semitone lower (i.e., pitch-incongruent sequence). We purposefully made the transposition subtle so that the off-pitch from the visual score was noticeable only by people with AP and not by those musicians who did not exhibit AP. For a third sequence, the auditory melody itself was different from the melody portrayed by the visual score (i.e., melody-incongruent).

The two rival targets were presented to separate halves of a 19-inch CRT monitor, which was used both in Korea University and Seoul National University (1024 x 768 resolution, 60-Hz frame rate; viewing distance 53 cm in KU, 60cm in SNU). The participant’s head was stabilized using a head/chin rest, and the auditory stimuli were delivered binaurally over headphones. Temporal synchrony of audio-visual stimuli was determined in a separate pilot test in which none of participants from the main experiment was tested. Beats per minute (bpm) of six auditory melodies were set to 95 quarter notes. The scroll speed of the visual musical scores was synchronized with the tempo of the auditory notes, with the reference point for synchronization being the middle of the checkerboard-textured frame. To achieve perfect synchrony, the visual musical scores streamed from right to left within the frame at the speed of 1.14°/s. The x/y position of the participant’s left eye was monitored at a sample rate of 500 Hz with a desk-mounted Eyelink 1000 (SR Research), controlled by Eyelink toolbox [43] provided in the Psychophysics toolbox.

We also conducted an AP test to categorize participants in terms of AP ability. On each trial of the test, participants heard a 2-s, computer-generated sine wave tone presented at a comfortable listening intensity over headphones. Pure-tone frequencies ranged from A2 to A6 (A4 = 442 Hz, concert-pitch), and each frequency was presented anywhere from one to three times. Participants then indicated the tone’s pitch, guessing if necessary, by clicking one of 12 piano keys (successive black and white notes ranging from C to B) displayed on a video monitor; participants were instructed to base their responses on pitch chroma, disregarding pitch height, and the visually displayed keyboard comprised just the 12 keys with no other context to suggest a particular octave placement along the 88-note keyboard. Immediately after making each response, participants heard a 1-sec presentation of white noise, inserted in the trial sequence so as to discourage use of the tone just heard as a reference for the next trial’s tone. Eighty tones in total were presented in a randomized order, and there was no imposed time limit for making a response following each tone presentation. We recognize that the absence of a response time limit could have helped people without AP by allowing them to work out their responses based on comparing what they heard relative to an internal, reference pitch they had memorized, such as the lowest pitch they could vocalize [19]. As a consequence of the unconstrained response time in our study, the performance of the non-AP individuals may have been inflated relative to their true AP ability, and this artifact could, in turn, reduce the likelihood of our finding a significant difference between AP and non-AP musicians. We revisit this point when discussing results of the AP test.

### Design and procedure

While viewing the visual stimuli through a mirror stereoscope and concurrently listening to a melody, participants used two keys on a computer keyboard to track periods of exclusive dominance during rivalry, the instruction being to press and hold a specified key for the duration of exclusive dominance of a specified rival target. Participants were instructed to maintain their fixation on the small fixation sign presented in the center of the images throughout the trial. Each trial lasted until the entire auditory melody had been presented, and the average duration of six melodies was 36.16 s (SD 5.07 s). In the analysis of dominance durations, the last duration was excluded if the button was being held down at the end of that trial; we did this to avoid including durations truncated by trial termination. Each visual score was repeated eight times and presented an equal number of times to each eye, yielding a total of 48 trials per participant. For accuracy of eye-tracking data, calibration was conducted at the start of every twelve trials using the standard five-point calibration procedure embedded in the Eyelink system controlled by Eyelink toolbox [43].

Each trial consisted of one of the three audio-visual (AV) conditions: “pitch-congruent”, “pitch-incongruent” (but melody congruent), or “melody-incongruent” (both pitch and melody incongruent) (Fig 1A right). We excluded a “no-sound” condition so as to concentrate trials distinguishing high- and low-level congruence specifically. On pitch-congruent trials (1/4 of trials), an auditory melody synchronized in time with the streaming visual score was heard over headphones. On pitch-incongruent trials (1/4 of trials), an auditory melody transposed from pitch-congruent trials was heard. Thus, audio-visual information presented on pitch-incongruent trials was congruent in terms of high-level symbolic association, but incongruent in regard to low-level acoustic feature-based multisensory integration, at least for AP group. Lastly on melody-incongruent trials (1/2 of trials), an auditory melody unrelated to the melodic lines of the visual score was heard. More specifically, a melody was randomly selected from five scores other than the one visually presented for melody-incongruent trials. In addition to this, one half of the melody-incongruent trials used the auditory melody in the original tune while the other half used the auditory melody transposed, although neither of them matched the synchronous visual musical score. The latter half of the trials was included to preclude more frequent presentation of specific melodies; we were concerned that such a mismatch might cause a (mis)understanding of the purpose of the experiment or influence rivalry dynamics.

In regard to the melody-incongruent trials, one might question the validity of using an auditory melody that corresponds to the score not being viewed, since it is completely nonmatching to the visual notations not only in pitch but also in pitch contour and rhythm of the notes. It is not impossible to create an auditory melody that retains contour and rhythm that match the visually presented score but does not match the score’s pitch. However, we were concerned about the impact on rivalry of the artificial quality of auditory melodies created in the way described above. Thus, rather than creating unnatural auditory stimuli for melody-incongruent trials, original melodies that follow the structures of conventional music were used. This also ensured that the number of times each melody was heard throughout the experiment was identical for all scores/melodies.

In the statistical analyses, we quasi-randomly sampled only a half of the melody-incongruent trials to match the number of trials in the other two conditions. Since each score/melody was presented twice either in pitch-congruent or in pitch-incongruent trials, the number of trials to be sampled from melody-incongruent trials was also limited to 2X for each score (one from melody-incongruent and the other from melody-incongruent and transposed trials). The 48 trials were broken into 8 blocks containing a mixture of pitch-congruent, pitch-incongruent, and melody-incongruent trials. Prior to formal data collection, participants were given a practice session of rivalry tracking between score and grating in the absence of sound. The AP test was administered following completion of the rivalry experiment, meaning that the experimenters had no knowledge about an individual’s AP ability during testing, with two exceptions (i.e., author participants SK and ML).

## Results

### AP test results

**Fig 2:**
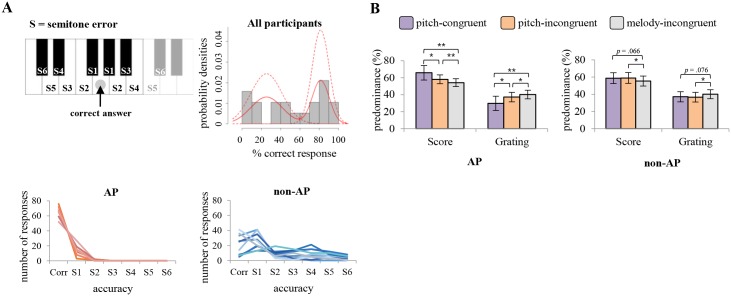
AP test results and predominance results. (A) AP test results. (Upper left) To indicate the pitch chroma of a brief tone heard through headphones, participants used a mouse to click on one of 12 piano keys presented on the monitor. S1-S6 denote the magnitudes of the semitone errors relative to the correct tone, irrespective of direction from the actual test frequency. (Upper right) The distribution of the percentage of correct answers fitted with the finite mixture model. Data of all participants are included. (Lower) Individual results from the AP test plotted for each group. Average percentages of correct answers were 81.53% (SD 9.33) for AP group and 25.13% (SD 17.16) for non-AP group. (B) Predominance values plotted for each group. Score predominance and grating predominance for each experimental condition do not necessarily add up to 100% due to the mixture perception. Error bars indicate 95% confidence interval. (*p < .05, **p < .01).

For each trial in the AP test, a participant’s response could be sorted into one of seven alternatives: a correct answer or any one of six possible incorrect answers defined in terms of tonal frequency distance from the correct key (Fig 2A upper left). Errors were not differentiated with respect to whether the semitone errors were upward or downward from the correct answer. We first calculated the percentage of correct answers for each participant, which ranged from 6.25% to 95% (5 to 76 correct answers out of 80, respectively). The distribution of the percentage of correct answers was fitted to the finite mixture model (see Fig 2A, upper right panel) using R equipped with mixtools [44, 45]. Examination of goodness of fit using a one-sample Kolmogorov-Smirnov test confirmed that the estimated mixture Gaussian distribution was not significantly different from the distribution of AP test scores (*p* = .96). The estimated cutoff of the mixture model was 67.13% (95% confidence interval 63.07% to 71.19%), thereby dividing participants into two groups. Accordingly, we deemed the nine people clustered in the high-score group as possessing AP and the other ten as being non-AP. Using a t-test to assess differences between the percentages of correct answers in the two groups thus defined, we found, not surprisingly, that scores were significantly higher for the AP group compared to the non-AP group (% correct answers *t* (14.161) = 9.017, *p* < .001, Cohen’s effect size index *d* = 4.792). Degrees of freedom were adjusted for the data since the Levene’s test on equality of variance was violated (*F* = 8.526, *p* = .01). That said, we pointed out earlier the possibility that some participants without AP may have produced inflated scores by using a response strategy that relied on relative pitch (e.g., comparing a heard tone to a memorized standard). This could be a contributing factor for the midrange scores seen in Fig 2A. It is worth noting that all the participants who scored in that midrange were categorized into the non-AP group.

We also examined the standard deviation in frequency units for responses on incorrect trials, to derive a scaled estimate of the degree of inaccuracy exhibited by each participant. This made it possible to examine qualitative differences in participants’ responses to pitch as well as the overall accuracy assessed by the number of correct answers. The graphs in the lower panel of Fig 2A summarize results from the analyses. The pattern of responses based on AP categorization is notably different when individual results are plotted separately for the two groups. Those whose accuracy score on the AP test placed them in the statistically defined AP group were also more accurate than non-AP people in discriminating pitch, and that was true for trials on which pitch was correctly identified and on trials producing an identification error. In other words, even when the pitch was incorrectly judged, the errors were nearly always within two semitones (S2) for the AP individuals, whereas the errors made by those in the non-AP category spanned the entire semitone range up to and including the largest possible value (S6). Interestingly, this grouping result is in line with unsolicited reports given by some participants after the binocular rivalry task: some of the individuals categorized as possessing AP reported that the pitch of a melody did not always match the accompanying, visual notation, but none of the participants within the non-AP group reported noticing such mismatches. Considered together, we are inclined to believe that our manipulations of pitch congruity would differentially impact AP and non-AP participants on the rivalry task, if rivalry is susceptible to congruence between pitch and visual notation. By way of preview, AP participants who commented on noticing that pitch was sometimes incongruent produced results on the rivalry task that were indistinguishable from results produced by AP participants who did not comment on incongruence.

### Predominance

Our initial focus of the analysis was on predominance, i.e., the total percentage of time a given rival target was experienced as exclusively dominant during a given observation period (Fig 2B). We first equalized the number of trials among the three conditions, since the melody-incongruent condition comprised twice as many trials compared to those in either pitch-congruent or pitch-incongruent conditions. Half of the trials were quasi-randomly sampled from the melody-incongruent trials, as described in the Methods. After balancing the number of trials for each sound condition, a two-way mixed ANOVA on score predominance values was performed with a within-subject factor of AV condition (pitch-congruent, pitch-incongruent, melody-incongruent) and a between-subject factor of AP possession (AP group vs. non-AP group). Because the results obtained with Mauchly’s test for sphericity failed to satisfy that assumption (x^2^ = 6.318, *p* = .042), the degrees of freedom were corrected with Greenhouse-Geisser estimates of sphericity (ε = .754). The main effect of AV condition and interaction effect between AV condition and group were significant (*F* (1.51, 25.64) = 13.632, *p* < .001, *n*_*p*_^*2*^ = .445, and *F* (1.51, 25.64) = 5.353, *p* = .018, *n*_*p*_^*2*^ = .239, respectively), but the main effect of group was not significant (*F* (1, 17) = .143, *p* = .71, *n*_*p*_^*2*^ = .008). These results imply that all participants in the current study were music readers and showed the general effect of audio-visual congruency on predominance regardless of AP possession, replicating the results from our previous work [17]. In further support of this implication, a paired *t*-test on AV conditions within group showed greater predominance for the musical scores over gratings when score was accompanied by an auditory melody that was congruent, either in pitch and notation (pitch-congruent) or in notation only (pitch-incongruent) compared to incongruent sound, and this was true in both the AP-group (*t* (8) = 3.857, *p* = .005, *d* = 1.115, and *t* (8) = 3.605, *p* = .007, *d* = 0.517, respectively) and the non-AP group except the marginally significant difference between pitch-congruent and melody-incongruent trials (*t* (9) = 2.096, *p* = .066, *d* = .345, and *t* (9) = 2.422, *p* = .038, *d* = .372, respectively).

Of paramount relevance to our current purpose, score predominance for pitch-congruent trials was significantly higher than score predominance for pitch-incongruent trials in the AP group (*t* (8) = 2.773, *p* = .024, *d* = .718), but not in the non-AP group (*t* (9) = -.16, *p* = .877, *d* = -.027). To reiterate, while there was a melodic congruence effect on predominance for both groups, the subtle pitch difference induced by transposition of congruent melody affected visual score predominance only for individuals who have an ability to recognize that subtle acoustic difference. Since the score predominance values from pitch-incongruent trials for both groups were comparable (58.02% in AP, and 59.02% in non-AP), it is tempting to characterize the effect of pitch congruence as an effect that is additive to the boost arising from melodic congruence (predominance: 65.74% in AP, and 58.75% in non-AP). Moreover, the equivalence of AP and non-AP score predominance values for pitch-incongruent trials suggests that differential degree of musical experience between the two groups is not a contributing factor to our results. Had the enhanced predominance in pitch-congruent trials observed only for the AP participants been somehow influenced by more musical training, e.g. a general tendency for a stronger coupling between audio-visual stimuli in musical context, group differences should have been observed in the predominance values of pitch-incongruent (but melody-congruent) trials as well. The absence of such an effect points to other factors as the bases of differences between performance measures of AP and non-AP musicians.

Lastly, we would like to address two points that deserve mention. First, the pattern of results found in the AP group was not influenced when we excluded the data of the two authors (who were among the AP group). One-way ANOVA with data from the remaining seven AP participants showed that the main effect of AV congruence remained statistically significant (*F* (1.17, 7.018) = 6.335, *p* = .037, *n*_*p*_^*2*^ = .514). Furthermore, results from *t*-tests performed on mean dominance scores between the AV conditions still revealed the same pattern of results whether the two authors’ data were included or not, although the consequence of reduced statistical power due to the reduced sample size was evident (pitch-congruent vs. pitch-incongruent: *p* = .08, *d* = 0.614; pitch-congruent vs. melody-incongruent: *p* = .031, *d* = 0.924; pitch-incongruent vs. melody-incongruent: *p* = .038, *d* = 0.413).

The second point to address stems from the possibility that participants scoring within the mid-range on the AP test were deploying an alternative strategy to perform the tone identification task. As already mentioned several times, participants without AP could score higher on the AP test by exploiting the absence of a response time limit to figure out pitch identity based on relative pitch. If this were the case, one would not expect differences between mid-range and low-range AP individuals on the binocular rivalry task, because pitch identification was not a requirement. However, when we performed the same statistical comparisons described above on score predominance values using three categories of participants, i.e. AP (N = 8), quasi-AP (N = 6) and non-AP (N = 5), we found a tendency for a stronger influence of AV congruency as a function of degree of AP ability (*F* (1.437, 22.997) = 9.115, *p* = .003, *n*_*p*_^*2*^ = .363; see S1 Fig). We surmise that this graded effect across groups may reflect the existence of a continuous distribution in AP ability, not a bimodal one [46–49].

### Dominance/Suppression durations

We next sought to identify the potential sources of the difference of predominance between trials when participants experienced congruence and trials when they experienced incongruence. Does the enhanced predominance of a visual score on the congruent trials result because a rival score remains dominant (i.e., for a longer duration), on average, or because it remains suppressed for a shorter duration, on average? Either of those alternatives, or a combination of both, could produce the enhanced predominance effect for the pitch-congruent trials in AP participants and on pitch-congruent and pitch-incongruent trials in non-AP participants. Here, we report rivalry results in terms of score dominance and score suppression durations. Just to be clear, each duration during which a score is exclusively visible constitutes a ‘score dominant’ event and, by definition, it also constitutes a ‘grating suppressed’ event; likewise, each duration of exclusive dominance of the vertical grating is also a ‘score suppressed’ event.

**Fig 3:**
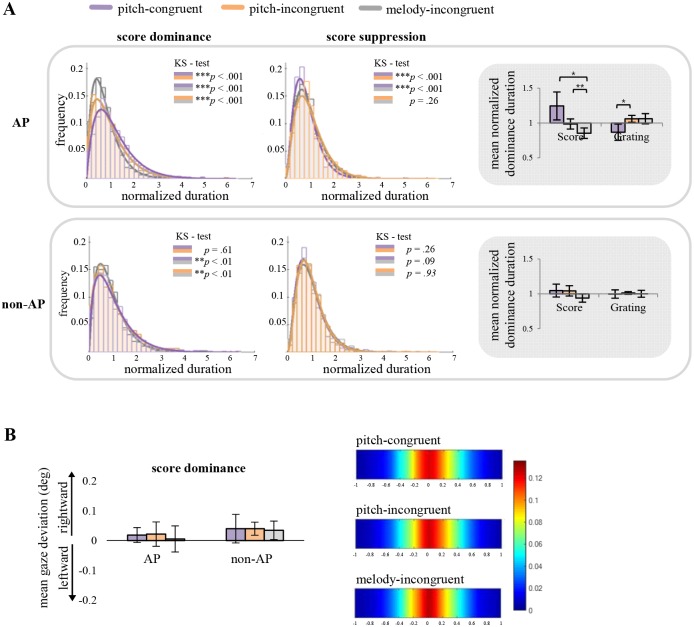
Dominance/Suppression durations and gaze-stability results. (A) Frequency histograms and best-fit gamma distributions of normalized dominance and suppression durations for the visual score. Histograms are constructed from data pooled across participants separately for AP and non-AP groups. The pairs of colored bars forming the KS-test insets show statistical likelihoods for pairwise comparisons using the Kolmogorov-Smirnov test. The pair of bar histograms on the right in each of the two panels show mean normalized durations for score dominance and score suppression (i.e. grating dominance) conditions. (B) Mean gaze-stability results from eye-tracking data. (Left) Mean gaze-stability during the score dominance durations plotted for each AP and non-AP groups. (Right) Heat-maps of mean gaze-stability results for each AV condition. Data of all participants are included in this analysis. The variable-color bar on the right side designates the relation between color and the incidence of shifts in fixation of different magnitudes relative to the exact point of fixation. Warm colors indicate stable fixation within +/- 0.2 deg of the fixation point.

As we expected based on other works [17, 50, 51], there were substantial individual differences in average dominance durations across participants (mean values ranged from 1.25–5.14 s). To allow us to pool data across those people forming a group, we first normalized the individual dominance/suppression durations of the scores for each individual. For normalization, the average of score dominance durations across all trials was calculated for a given individual (e.g., [52]), and the resulting value was used to divide each dominance duration. By doing this, we expected to minimize across-participants difference that comes from individual’s general tendency of how long one stimulus stays dominant before alternation to the other stimulus. The same normalization was performed separately for score suppression duration. A two-way mixed ANOVA on normalized dominance durations showed that the main effect of AV condition was significant (*F* (1.307, 22.224) = 8.307, *p* = .005, *n*_*p*_^*2*^ = .328) whereas the main effect of group and interaction between two factors did not reach significance (*F* (1, 17) = 1.255, *p* = .278, *n*_*p*_^*2*^ = .069, and *F* (1.307, 22.224) = 3.168, *p* = .079, *n*_*p*_^*2*^ = .157, respectively, see the insets on the right in Fig 3A). As in the analysis on predominance values, we additionally analyzed the data based on a three-group categorization, this time focusing on normalized dominance/suppression durations. Again, the boost in dominance durations associated with AV congruence was greater for those who scored high on the AP test (S2 Fig), with the only statistically significant result being a main effect of AV condition (*F* (1.294, 20.706) = 5.57, *p* = .021, *n*_*p*_^*2*^ = .258).

Further comparisons on normalized score dominance durations of the AP group followed the same pattern observed in predominance. Specifically, the differential congruence effect for the AP group stemmed from the overall longer dominance durations of scores on melody-congruent (both pitch-congruent and pitch-incongruent) trials compared to melody-incongruent trials (pitch-congruent vs melody-incongruent: *t* (8) = 3.014, *p* = .017, *d* = 1.736, pitch-incongruent vs melody-incongruent: *t* (8) = 3.723, *p* = .006, *d* = 1.517). A novel, revealing finding was that normalized score suppression durations for pitch-congruent trials were shorter than those for pitch-incongruent trials (*t* (8) = -2.607, *p* = .031, *d* = -1.415). In other words, a visual score tended to be released from suppression more quickly when accompanied by pitch-congruent melody, compared to when accompanied by congruent but transposed melody. This suggests that congruence/incongruence defined by the relatively low-level auditory property of tone frequency modulated the effective strength of a suppressed visual stimulus in people who are highly sensitive to absolute pitch.

For the non-AP group, we observed that normalized dominance durations yielded a similar pattern of congruence effect as that observed within the AP group. However, the statistical result failed to reach significance, leading to further analysis only with two AV trials–congruent vs. incongruent trials. Having shown that the pitch-congruent and pitch-incongruent sounds are experienced as virtually the same for non-AP group, we reasoned that collapsing those trials into congruent trials to be compared to incongruent trials (including all, not half) would compensate for an insufficient number of trials that could have compromised statistical power. With these pooled data, results showed that normalized dominance durations for congruent trials were indeed significantly longer than those of incongruent trials (*t* (9) = 2.766, *p* = .022, *d* = 1.744). Turning to the normalized suppression durations, no significant differences between AV conditions were found in the non-AP group. This, of course, is different from what we found for the AP group.

This pattern of results can be seen in the frequency histograms of normalized durations for the three conditions pooled across participants (Fig 3A). As routinely seen in other rivalry studies [5, 53–57], those histograms for all three AV conditions conform closely to the gamma distribution, for both AP and non-AP participants. But notice, too, the differences in the peaks of those distributions, suggestive of an impact of audio-visual congruence on rivalry durations. To statistically evaluate whether those apparent differences between distributions are significant, we performed pair-wise Kolmogorov-Smirnov tests on the normalized dominance/suppression durations, for each pair of AV conditions. Considering first the normalized dominance durations of the AP group, distributions for pitch-congruent vs pitch-incongruent trials were significantly different from one another (two-sample Kolmogorov-Smirnov test *p* < .001, d = .112). This was also true when the distribution of normalized dominance durations for melody-incongruent trials was compared to that of pitch-congruent and pitch-incongruent trials, respectively (*p* < .001, d = .215; *p* < .001, d = .124, respectively). Considering next the non-AP group, statistically significant differences were found between distributions of melody-congruent and melody-incongruent trials (*p* = .003, d = .09; *p* = .009, d = .08, for melody-incongruent trials compared to pitch-congruent or pitch-incongruent trials respectively) but not between pitch-congruent and pitch-incongruent trials (*p* = .61, d = .037). That is, pitch congruity between sound and musical score resulted in a higher incidence of longer dominance durations only among participants able to detect a subtle difference in auditory pitch and visual notation. When the score is suppressed, fitted lines are essentially superimposed and, thus, indistinguishable for the non-AP groups. On the other hand, there is a difference in frequency between conditions marked by higher frequency of shorter durations in pitch-congruent trials for AP group. This is again supported by two-sample Kolmogorov-Smirnov tests, showing significant difference between pitch-congruent and either of the other two AV conditions only in AP group (*p* < .001, d = .14; *p* < .001, d = .1, when compared to pitch-incongruent or melody-incongruent trials respectively, *p* = .26, d = .05, for pitch-incongruent and melody-incongruent trials).

### Eye movement data

We recorded participants’ eye movements while they tracked binocular rivalry. Even though participants were instructed to maintain central fixation, it was possible that the rival stimuli—i.e., a musical score and a grating scrolling laterally in opposite directions—could destabilize fixation by inducing horizontal eye movements [54]. We thus felt it necessary to determine whether differences existed in these reflexive eye movements across the AV conditions, for those differences could influence the pattern of perceptual dynamics associated with those conditions. We calculated the mean deviations of horizontal eye movements from the fixation point for each AV condition, separately for each person in the AP group and in the non-AP group. Owing to the technical problems with the eye tracking system, data from one AP participant and one non-AP participant had to be excluded.

When values of mean gaze deviations were entered into repeated-measures ANOVA, neither the main effect of the AV congruence nor group was significant (*F* (1.419, 21.279) = .778, *p* = .431, *n*_*p*_^*2*^ = .049; *F* (1, 15) = .778, *p* = .431, *n*_*p*_^*2*^ = .049) nor was the interaction between those two factors (*F* (1, 15) = 2.687, *p* = .122, *n*_*p*_^*2*^ = .152) (Fig 3B, left). Since gaze stability in the two groups was equivalent, data for the two groups for each AV condition were combined to assess possible eye stability differences for the AV conditions. No differences were revealed among those conditions (one-way ANOVA, *F* (1.427, 22.826) = .778, *p* = .431, *n*_*p*_^*2*^ = .046), and the results from that analysis is shown in the form of stability heat maps in Fig 3B, right. It is highly unlikely therefore that eye movement differences contributed to the discernable pattern of score dominance under the different AV conditions.

## Discussion

In the current study, we observed several influences of congruence on binocular rivalry dynamics, one replicating an earlier finding and the other revealing a novel bisensory interaction.

Replicating Lee et al. [17], we again found that a musical score enjoyed enhanced predominance during rivalry when that score was accompanied by an auditory melody that was congruent with the score. This was true for all musically trained participants, both those with AP and those showing no evidence for AP. Moreover, this boost in predominance was realized as a consequence of lengthened dominance durations for the melodically congruent score, not by abbreviated suppression durations of the score. This, too, replicates our earlier findings.

In addition, we also discovered that pitch congruence between score and auditory melody further enhanced score predominance, relative to the pitch-incongruent/melody-congruent condition, but only in AP participants. This double boost in predominance from pitch and melody congruence enjoyed by AP participants is perhaps not so surprising, because the two sources of congruence are quite different in origin: pitch congruence is defined by correspondence between tone frequency and visual designation of a musical note–that correspondence can be realized in musical passages, non-musical passages or single tone/note pairings (as used in the AP test). Melodic correspondence represents a more abstract form of comparison that can only be realized by the unfolding sequence of notes seen and heard, and that correspondence generalizes across transpositions in note positions in the auditory dimension or in the visual notational dimension.

Another novel finding from the current study is that the melody perfectly matching the visual score affected score suppression durations in AP participants: pitch-congruent visual notations emerged from suppression (i.e., regain visual dominance) sooner than did incongruent melodies. At the same time, there was no difference in suppression durations between the pitch-incongruent and melody-incongruent trials, and that was true for both AP and non-AP participants. Evidently, then, there is something unique about pitch information that allows it to potentiate the visual salience of a visual score for people who can sense pitch congruence. Pitch, unlike melody, is a stimulus property associated with acoustic frequency, itself a low-level auditory sensory quality. Viewed in this way, it is perhaps not surprising that people who can discern absolute pitch information are sensitive to the congruence of pitch and melody of a visually suppressed line of score. In other rivalry studies, it has been found that acoustic signals [15, 58, 59] and tactile stimuli [13, 14] can abbreviate suppression durations of a visual stimulus paired with those auditory or those tactile events if the multisensory events are congruent. In this vein, the more rapid transition from suppression to dominance found for visual musical scores paired with pitch-congruent melody likely stems from low-level acoustic features (i.e., tonal frequency) defining pitch-congruence, not just the congruent melody line accompanying melody-congruence alone.

Accompanying the remarkable perceptual ability to precisely discern the pitches of musical notes are unusual patterns of brain activation within diverse cortical networks. It is beyond the scope of our paper to delve into the details of this large, growing literature, but we will single out just a few highlights that might bear on our findings. One generality that emerges is an association between AP and activations within the dorsolateral prefrontal cortex (DLPFC), which is thought to play a role in formation and retrieval of associate memories [31, 60–63]. Because pitch labeling entails associating an arbitrary note designation and a pitch chroma, AP might be related to an exceptional ability to retrieve a pitch memory that is characterized not only by distinct functional activity in DLPFC [47, 64] but also by significantly different brain anatomy in that area, including cortical thickness [65]. Another area implicated in AP is the planum temporale (PT), a region associated with auditory processing and language. Hemispheric differences in PT size are more pronounced in people with AP compared to non-AP individuals [60, 66], with the volume of the left PT area being positively correlated with pitch naming task performance [31]. Of particular relevance to the current work, the PT brain area appears to respond to multisensory inputs [23, 67], and the left PT can be activated in trained musicians simply by watching the hands of someone else playing piano keys [68]. Given that PT is involved in audio-visual processing and that its activation is correlated with AP ability, it is tempting to wonder whether PT contributes to the more pronounced impact of audio-visual congruence on visual selection during binocular rivalry among those who possess AP. These studies just mentioned as well as others [66–70] reveal that within the brains of at least some musicians there exist robust cross-modal interactions between hearing and seeing musical notes, interactions that could selectively enhance the visual strength of musical scores accompanied by congruent melodies evidenced in all musically trained participants, AP and non-AP alike.

We are also intrigued by the possibility that auditory-evoked imagery might play a role in the results found in our study. Zatorre and Beckett [71] have speculated that auditory pitch can be encoded by people with AP through the engagement of visual imagery. Based on their suggestion and the neural evidence supporting it [31], we can envisage that visual imagery triggered by *hearing* an auditory melody might contribute to the enhanced rivalry dominance of that melody’s congruent visual score. After all, we know from previous studies that merely imagining a given visual contour orientation [72] or a specific color [73] can bias subsequent dominance of that particular stimulus property when the actual stimulus is engaged in binocular rivalry. We see no reason why visual imagery of a score could not also be evoked by hearing an auditory melody. Recall, too, that our findings also indicate that visual scores suppressed from awareness emerge from suppression more quickly (i.e., score suppression durations are briefer) when an invisible score and the accompanying auditory melody are congruent. This is why subliminal impact becomes easier to interpret within the context of auditory-evoked imagery, for the imagined note sequence could serve to boost the strength of the neural representation of the suppressed score and, thus, abbreviate suppression durations in the same way that increases in the actual strength of a suppressed visual stimulus hastens its return to dominance [74–77]. In this context, we note again that sensory signals in other modalities [12–16, 58, 59] exhibit effects on suppression durations similar to those we have found for auditory pitch in AP participants.

Lastly, in regard to the aforementioned distinction of the pitch-incongruent trials from the pitch-congruent and the melody-incongruent trials, it is worth considering our results within the context of a predictive coding account of rivalry [76, 78]. According to predictive coding, perception is jointly determined by sensory data and by the internal prediction with the best explanation ability [78]. It is assumed that the ultimate goal of perceptual neural processing is to minimize prediction errors and free-energy by deciding the prediction that explains the sensory input to the greatest extent. As Hohwy et al. [76] discussed, selection and alternation of the visual stimuli during binocular rivalry can also be accounted for by the dynamic weighing on the estimated likelihoods of two possible predictions. Furthermore, the prediction temporarily selected as the most viable visual interpretation can be influenced by cross-modal information [79, 80]. Thus in our study, auditory melody might have provided a constraint on the plausibility of two visual predictions, one for the musical score and the other for the grating. Transposed melodies presumably lowered the likelihood of the visual interpretation in favor of the musical score as a current percept for AP possessors, delaying the temporary emergence of dominance of visual score over the grating. On the other hand, in the pitch-congruent condition, an auditory sound perfectly matching the viewed score might hasten the decision of dominance by biasing likelihood in favor of score dominance over grating dominance. This interpretation, of course, could be construed simply as a restatement of the actual results, cast in a statistical framework [81]. Until this account can be quantitatively tested along the lines employed in other multisensory studies (e.g., [80]), it remains pure conjecture. What is not conjecture, however, is the existence of potent multimodal context effects in the unfolding dynamics of binocular rivalry involving visual and auditory musical events.

## Supporting information

S1 Fig Predominance. Predominance values plotted for each group based on three-group categorization. Asterisks indicate statistically significant difference between a pair of AV conditions (* *p* < .05).
(TIF)

S2 Fig Normalized dominance/suppression durations. Mean values of normalized dominance and suppression durations plotted for each group based on three-group categorization. Asterisks indicate statistically significant difference between a pair of AV conditions (* *p* < .05).
(TIF)
