# Supplementary figures and images for: Audio-visual interactions uniquely contribute to resolution of visual conflict in people possessing absolute pitch
Source: PLoS One. 2017 Apr 5;12(4):e0175103. doi: 10.1371/journal.pone.0175103 (PMC5381860; doi:10.1371/journal.pone.0175103)

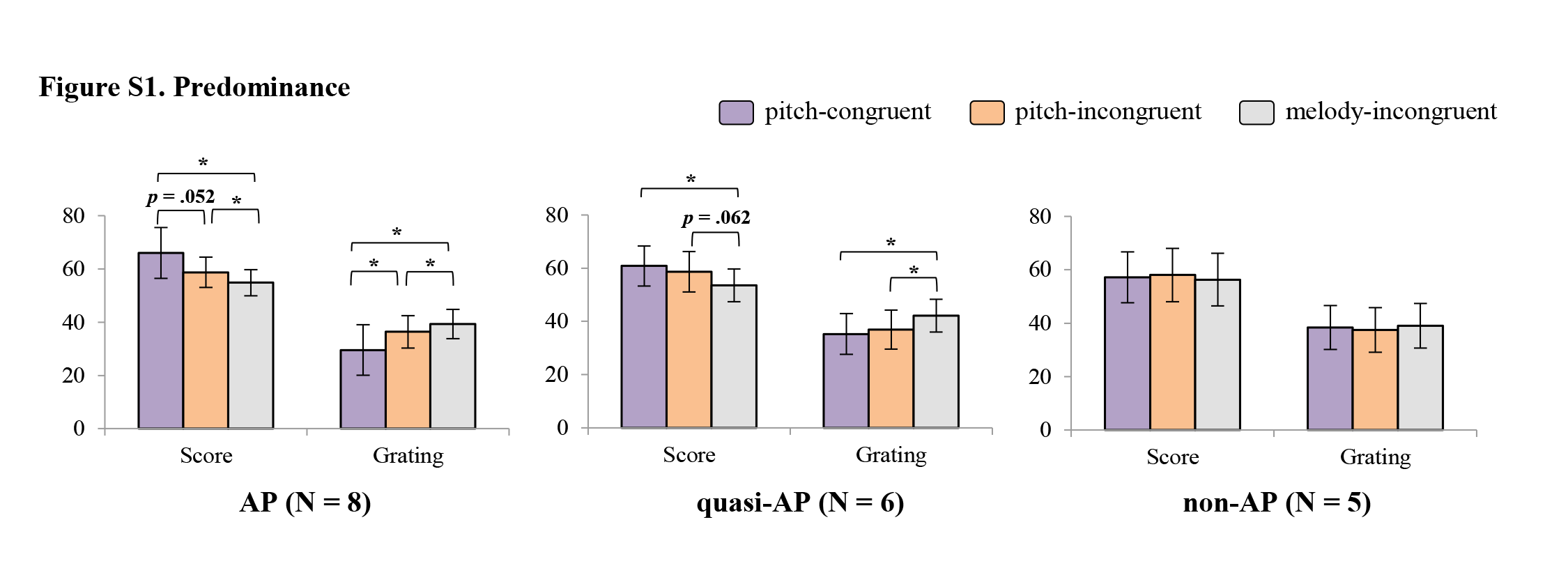

Supplement: S1 Fig — Predominance values plotted for each group based on three-group categorization. Asterisks indicate statistically significant difference between a pair of AV conditions (* p < .05). (TIF) [file pone.0175103.s001.tif]

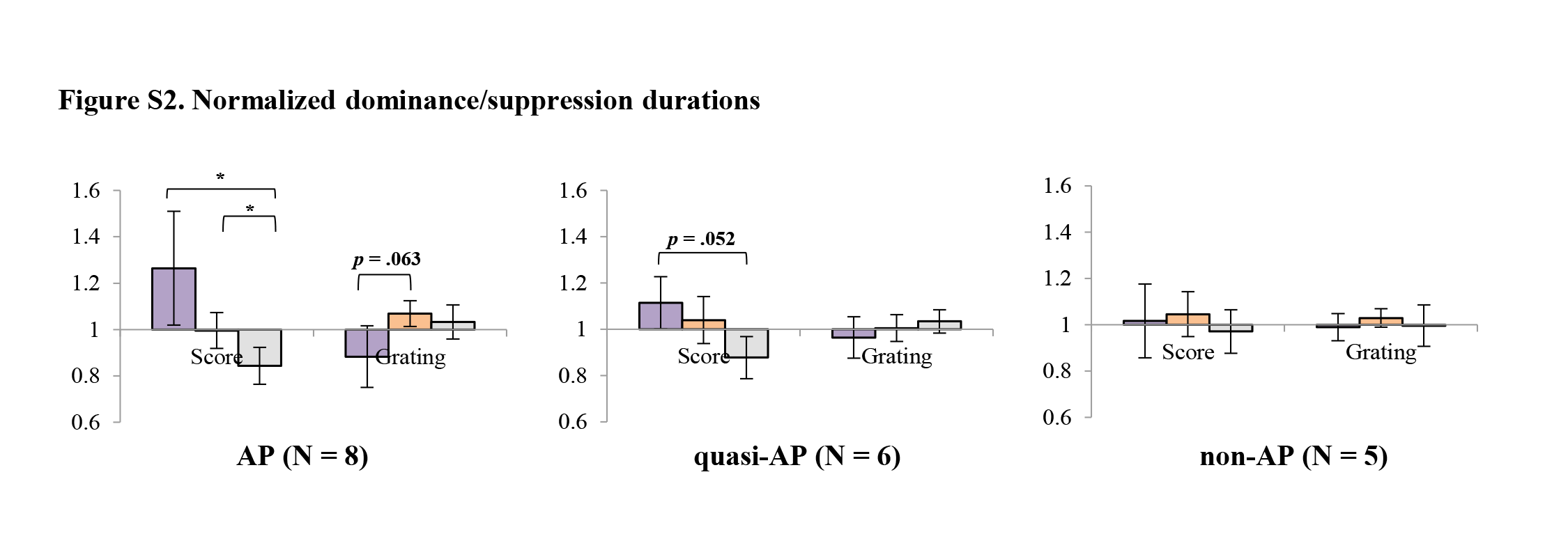

Supplement: S2 Fig — Mean values of normalized dominance and suppression durations plotted for each group based on three-group categorization. Asterisks indicate statistically significant difference between a pair of AV conditions (* p < .05). (TIF) [file pone.0175103.s002.tif]
